# Anti-Inflammatory Effects of Hydroethanolic Extract from *Ehretia asperula* on Lipopolysaccharide-Stimulated RAW264.7 Macrophages

**DOI:** 10.4014/jmb.2403.03006

**Published:** 2024-04-15

**Authors:** Bao Le, Vo Thi Kim Hong, Seung Hwan Yang

**Affiliations:** 1 Faculty of Pharmacy, Ton Duc Thang University, Ho Chi Minh City 72915, Vietnam; 2 Department of Biotechnology, Chonnam National University, Yeosu, Chonnam 59626, Republic of Korea

**Keywords:** Cyclooxygenase, cytokine, inflammatory, nitric oxide, phagocytosis

## Abstract

*Ehretia asperula* is a medicinal plant of the *Ehretia*ceae family used to treat inflammatory disorders, but the underlying mechanisms are not fully elucidated. The anti-inflammatory potential was determined based on enzyme cyclooxygenase-2 (COX-2) inhibition, which showed that the 95% ethanol extract (95ECH) was most effective with a half-maximal inhibitory concentration (IC_50_) value of 34.09 μg/mL. The effects of 95ECH on phagocytosis, NO production, gene, and protein expression of the cyclooxygenase 2/prostaglandin E2 (COX-2/PGE2) and inducible nitric oxide synthase/nitric oxide (iNOS/NO) pathways in lipopolysaccharide (LPS)-induced RAW264.7 cells were examined using the neutral red uptake and Griess assays, reverse-transcriptase polymerase chain reactions (RTPCR), and enzyme-linked immunosorbent assays (ELISA). The results showed that 95ECH suppressed phagocytosis and the NO production in activated macrophage cells (*p* < 0.01). Conversely, 95ECH regulated the expression levels of mRNAs for cytokines tumor necrosis factor alpha (TNF-α), interleukin-6 (IL-6), and interleukin-1 beta (IL-1β) as well as the corresponding proteins. In addition, PGE2 production was inhibited in a dose-dependent manner by 95ECH, and the expression of iNOS and COX-2 mRNAs was decreased in activated macrophage cells, as expected. Therefore, 95ECH from *E. asperula* leaves contains potentially valuable compounds for use in inflammation management.

## Introduction

Inflammation is a response that occurs via complex pathological processes against numerous external injurious and pathological events [1]. Macrophages are a pivotal component of the first line of innate defense against microbial infection, injury, or endogenous biomolecules. In the defensive response, nitrite oxide (NO) and cyclooxygenase-2 (COX-2) are two major products of activated macrophages that enhance various intrinsic mechanisms, including producing pro-inflammatory cytokines, such as tumor necrosis factor α (TNF-α), interleukin-1 beta (IL-1b), and interleukin-6 (IL-6) [2]. Although numerous effective therapeutic drug strategies are available, inflammation remains at the forefront of public health research owing to the diverse array of adverse effects and immunomodulator discontinuation [3]. Based on an increasing understanding of medicinal plants, novel natural compounds have been developed and have shown synergy with various therapies in clinical trials.

The *Ehretia* genus belongs to the family Boraginaceae and comprises approximately 150 species extensively dispersed in Asia [4]. Among them, *Ehretia asperula* has been widely used for thousands of years as a remedy for inflammation in traditional Vietnamese medicine to treat various diseases, including stomach disorders, infectious diseases, and arthritis. Phytochemical investigations have shown that bark and leaves from the genus *Ehretia* are sources of diverse metabolites, including benzoquinones, flavonoids, alkaloids, and cyanogenetic glycosides [5, 6]. These compounds have demonstrated strong antioxidant, anti-inflammatory, and antiarthritic effects. The anti-inflammatory potential of metabolites isolated from *E. dicksonii*, *E. laevis*, and *E. acuminata* has also been investigated in vitro [4, 7-9]. In addition, the methanol extract from *E. cymosa* leaves induces anti-inflammatory effects and inhibits the development of carrageenan-induced paw edema [7]; however, this activity is poorly studied in other species.

In the present study, we evaluated the anti-inflammatory effects of different extracts of *E. asperula* via inhibition of COX-2 activity in vitro. We then selected the most effective extract and investigated its role in inflammatory-activated RAW264.7 cells through inhibition of NO production and phagocytosis to reveal the mechanisms underlying its traditional use for inflammation management. The findings may be of great significance in validating the folkloric use and functional food development of the genus *Ehretia* in regulating inflammation.

## Materials and Methods

Folin-Ciocalteu’s phenol reagent, gallic acid, rutin, lipopolysaccharide (LPS) from i serotype enteritidis, neutral red solution, and Griess reagent (modified) were purchased from Sigma Chemical (USA). A COX-2 (human) Inhibitor Screening Assay Kit was purchased from Cayman Chemical (#701080, Ann Arbor, USA). An EZ-Cytox Cell Viability Assay Kit was purchased from Daeil Lab Service (Republic of Korea). TNF-α, IL-1β, IL-6, and PGE2 were purchased from R&D Systems (USA). Dulbeccós modified Eaglés medium (DMEM, #LM001-7), heat-inactivated fetal bovine serum (FBS, # S101-01), and penicillin-streptomycin solution (LS202-02) were purchased from WelGENE (Republic of Korea). Quick Start Bradford Protein Assay (#5000205), PureZOL RNA Isolation Reagent (#7326880), iScript cDNA Synthesis Kit (#1708890), and iQ SYBR Green Supermix (#1708880) were purchased from Bio-Rad Laboratories (USA). The mouse-derived RAW264.7 macrophage cell line was obtained from the American Type Culture Collection (ATCC, USA).

### Preparation of *E. asperula* Leaf Extracts

*E. asperula* leaves were collected in Bac Giang Province, Viet Nam, and were washed with running water. The leaves were air-dried for 20 h in a 40°C oven with ventilation (UN55, Memmert, Germany) before being pulverized using an electric grinder. The dried leaf powder (10 g) was soaked in 100 ml of different types of solvents (hexane, methanol, 95% ethanol, 80% ethanol, 50% ethanol, and water) in a rotary shaker at room temperature for 24 h. Each extracted solvent was filtered using a vacuum filtration apparatus equipped with a pump (N 820 FT.18, KNF Neuberger, USA) and 0.45 μm filter paper. The solvent was fed into a rotary evaporator (R-100, BUCHI Labortechnik AG, Switzerland) to obtain crude extracts. In all cases, the filtrate was freeze-dried at −80°C using a freeze dryer (ALPHA 2-4 LD Plus, , Germany) to obtain the freeze-dried hexane extract (n-HCH), methanol extract (MCH), 95% ethanol extract (95ECH), 80% ethanol extract (80ECH), 50% ethanol extract (50ECH), and aqueous extract (WCH). The extraction yield represents the percentage of the dry weight after the preparation procedure. The voucher specimens have been deposited at Ton Duc Thang University and Chonnam National University.

### Determination of Total Phenolic and Flavonoid Contents

To estimate the total phenolic content (TPC), 0.50 ml of sample solution and 0.125 ml of Folin-Ciocalteu reagent were incubated at room temperature in the dark for 5 min. Next, 1.25 ml of 7% Na_2_CO_3_ was added, and the mixture was further incubated under the same conditions for 20 min. The absorbance was then recorded at 765 nm using a UV/Vis spectrophotometer (UV-2550, Shimadzu, Japan). The TPC of the sample was calculated using a gallic acid standard curve and expressed as milligrams of gallic acid equivalents per gram of extract (mg GAE/g extract).

To determine the total flavonoid content (TFC), the sample solution was mixed with 0.6 ml of 5% NaNO_2_. The reaction mixture was mixed with 0.5 ml of Al(NO_3_)_3_ (10% w/v) and analyzed using a UV/Vis spectrophotometer at 430 nm to determine the absorbance. The TFC of the sample was calculated using a rutin standard curve and expressed as milligrams of rutin equivalents per gram of extract (mg RE/g extract).

### In Vitro Determination of COX-2 Inhibition Activity

A COX-2 inhibitory screening assay kit was used to assess the potential inhibition of human COX-2 by extracts. The amount of prostaglandin F2α (PGF2α) generated from arachidonic acid (AA, C20:4 [*n*-6]) via cyclooxygenase reaction was determined in the presence of extract (at 7.5-480 μg/ml) or DMSO. Subsequently, the amount of PGF2α produced was quantified via ELISA using an antiserum specific to PGF2α with absorbance measurement at 405 nm.

### Cell Preparation and Viability Test

Mouse-derived RAW264.7 macrophages were grown under humidity conditions at 37°C and 5% CO_2_. DMEM with 4500 mg/l D-glucose supplemented with 10% inactivated FBS and 1X penicillin/streptomycin was used for all cell experiments. Cell viability was evaluated using the EZ-Cytox Cell Viability Assay Kit according to manufacturer’s instructions. Briefly, 1 × 10^4^ cells were seeded into each well of 96-well plates and incubated overnight. Then, the culture medium was removed, and the cells were treated with fresh medium without or with 1 μg/ml LPS and various concentrations of extract. After incubation for 24 h, 10 μl of WST-1 reagent was added to each well and followed by incubation for an additional 4 h. After that, the plate was placed on ice for 10 min to stop the reaction. Absorbance values at 450 nm were measured using a microplate reader. The cell viability of the control group treated with DMEM was set as 100%.

### Determination of Phagocytosis

The neutral red uptake procedure was performed as previously described [10]. In brief, seedlings (1 × 10^5^ RAW264.7 cells) were allowed to adhere for 4 h in 12-well plates with DMEM (10% FBS). Cells were washed with cold phosphate-buffered saline (PBS) and then incubated in fresh DMEM supplemented with or without LPS (1 μg/ml) and various concentrations of extract for a further 24 h. After incubation, cells were washed with PBS and 200 μl of 0.33% neutral red solution was added to each well for another 1 h. The non-phagocytized neutral red was removed by PBS, and 200 μl of lysis solution (acetic acid: alcohol = 1:1 *v/v*) was added and the mixture was incubated for 1 h at 37°C to dissolve the neutral red. The resulting optical density was measured at 540 nm using a microplate reader and the cell phagocytosis rate was calculated using the following equation:

$$Cell\;phagocytic\;rate(\%)=\frac{Ab-Ao}{Ac-Ao}\times 100$$ [1]

where A_b_ is the absorbance of the sample, A_o_ is the absorbance of the blank (without sample), and A_c_ is the absorbance of the control group.

### Determination of Nitric Oxide Production

The Griess procedure was performed as previously described [11]. In brief, seedlings (1 × 10^5^ cells) were allowed to adhere for 4 h in 12-well plates. Cells were washed with cold PBS and then incubated in fresh DMEM supplemented with or without LPS (1 μg/ml) and various concentrations of extract for a further 24 h. The cell supernatant was collected and mixed with 100 μl Griess reagent for 10 min. The resulting optical density was measured at 545 nm using a microplate reader and nitrite concentration was calculated with standard curves of sodium nitrite (NaNO_2_).

### Determination of Cytokine and Prostaglandin E2 Production

To determine pro-inflammatory cytokine secretion, 50 μl of cell supernatant was collected to measure the concentrations of TNF-α, IL-1β, IL-6, and PGE2 in accordance with the murine ELISA kit instructions. The protein contents were assessed using Quick Start Bradford Protein assays with bovine serum albumin (BSA) as the standard.

### Real-Time Reverse Transcription Polymerase Chain Reaction (RT-PCR)

Total RNA from RAW264.7 cells was isolated using the PureZOL RNA Isolation reagent according to the manufacturer's protocol. To synthesize cDNA, RNA (2 μg/ μl) was mixed with a master mix of the iScript cDNA Synthesis Kit in a 20 μl reaction. The target genes were amplified and quantified using the iQ SYBR Green Supermix in a Rotor-Gene Q (Qiagen, Japan). The primers of treated cells were as follows: TNF-α-F: ATCCAT CTCTTTGCGGAGGC; TNF-α-R: GGGGGAGAGGTAGGGATGTT; IL-1β-F: TGCCACCTTTTGACAGTGATG; IL-1β-R: TGATGTGCTGCTGCGAGATT; IL-6-F: AGTTGCCTTCTTGGGACTG; IL-6-R: TCCACGATTTCC CAGAGAACG; COX2-F: AGAGGTAATCCAGACTCTGCT; COX2-R: TGCTCATACATTCCCCACGG; iNOS-F: CCCTTCCGAAGTTTCTGGCAGCAGC; iNOS-R: GGCTGTCAGAGCCTCGTGGCTTTGG; GAPDH-F: GGCCTCCAAGGAGTAAGGTC; and GAPDH-R: AGATTCTCAGTGTGGCGGAG. GAPDH was used as an internal reference gene and normalized using the 2^−ΔΔCt^ method [12].

### Statistical Analysis

All data were analyzed in GraphPad Prism 9.0 and statistical significance was set at either *p* < 0.05, *p* < 0.01, or *p* < 0.001. The IC_50_ of extracts against the COX-2 enzyme was calculated using the “Log (inhibitor) vs. normalized slope of response variable” method with GraphPad Prism 9.0.

## Results

### Chemical Composition of *E. asperula* Leaf Extracts

There were significant differences (*p* < 0.05) in the yield of *E. asperula* leaf extracts, which varied from 5.3 to 29.8% depending on the type of solvent and growing conditions. The yields of the hexane, methanol, 95% ethanol, 80% ethanol, 50% ethanol, and water extracts were 5.3 ± 0.3% (w/w), 14.0 ± 0.2% (w/w), 29.8 ± 0.3% (w/w), 16.7 ± 0.3% (w/w), 10.1 ± 0.3% (w/w), and 15.6 ± 0.3% (w/w), respectively.

The qualitative analytical results of total phenolic and flavonoid contents are illustrated in Table 1. Each gram of *E. asperula* leaves had a phenolic content of up to 156.12 mg GAE, as well as a flavonoid content of up to 91 mg RE. The 95% ethanol was the most efficient solvent to recover phenolic content from *E. asperula* leaves, followed by 80% ethanol, hexane, methanol, 50% ethanol, and water. The TFCs from *E. asperula* leaves extracted using 95%ethanol, hexane, 80% ethanol, 50% ethanol, water, and methanol were 91.65, 90.58, 62.25, 60.31, 40.69, and 39.25 mg RE per gram, respectively.

### Inhibitory Activity of *E. asperula* Leaf Extracts

**Fig. 1:**
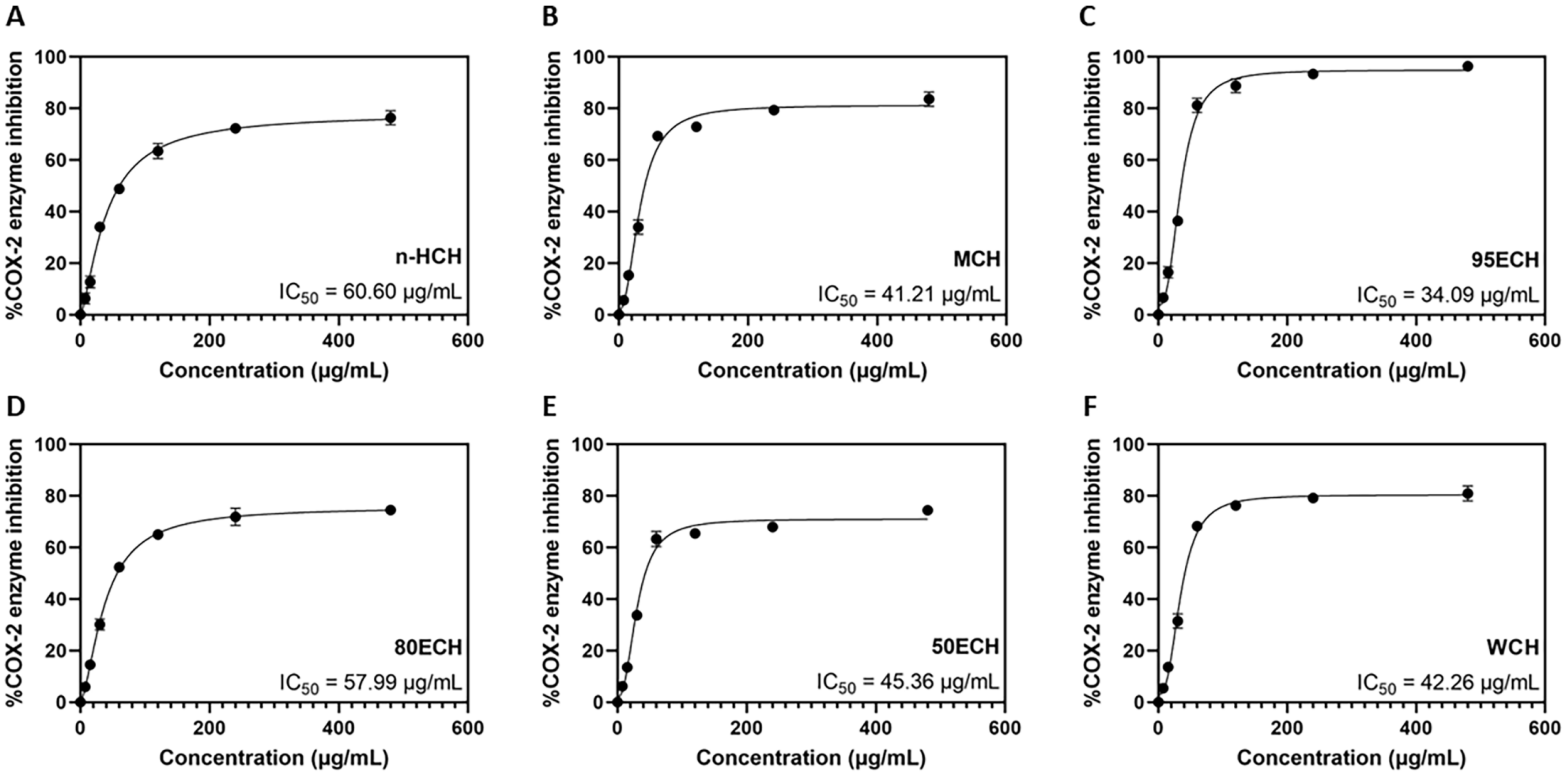
Inhibitory activity of *E. asperula* leave extracts against cyclooxygenase 2 enzyme. (**A**) IC_50_ curves of nhexane extract; (**B**) IC_50_ curves of methanol extract; (**C**) IC_50_ curves of 95% ethanol extract; (**D**) IC_50_ curves of 80% ethanol extract; (**E**) IC_50_ curves of 80% ethanol extract; and (**F**). IC_50_ curves of aqueous extract. n-HCH, hexane extract; MCH, methanol extract; 95ECH, 95% ethanol extract; 80ECH, 80% ethanol extract; 50ECH, 50% ethanol extract; WCH, aqueous extract; COX- 2, cyclooxygenase-2; IC_50_, half-maximal inhibitory concentration.

The anti-inflammatory effects of *E. asperula* leaf extracts were evaluated using a commercial kit for screening for human COX-2 inhibition. Our findings indicate that *E. asperula* leaf extracts inhibited COX-2 in a concentration-dependent manner with high selectivity for 95ECH. The half-maximal inhibitory concentrations (IC_50_) of MCH, WCH, 50ECH, 80ECH, and n-HCH were 41.21, 42.26, 45.36, 57.99, and 60.60 μg/ml, respectively (Fig. 1). These results suggest that the 95ECH of *E. asperula* leaves had anti-inflammatory potential, and thus it was selected for further analysis.

### Effects of 95ECH from *E. asperula* Leaves on Activated RAW246.7 Cells

**Fig. 2:**
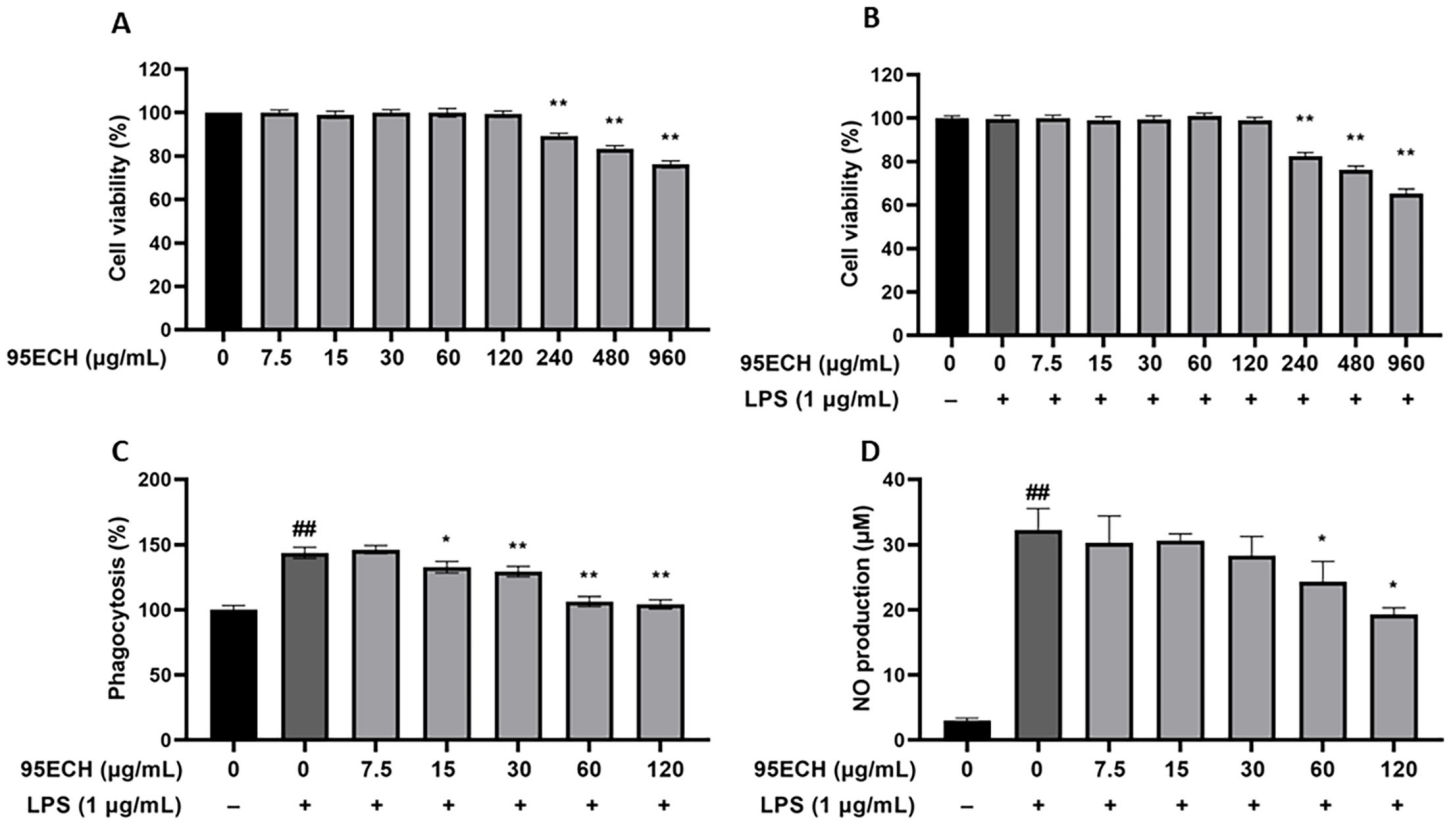
Effects of 95% ethanol extract of *E. asperula* leaves on the cell viability of RAW264.7 cells without LPS. (**A**) Cell viability of RAW 264.7 cells with LPS; (**B**) phagocytosis (**C**), and NO production (**D**). ^##^*p* < 0.01 represents a significant difference compared with the control group; *p < 0.05, **p < 0.01 represent a significant difference compared with the LPS alone group. 95ECH, 95% ethanol extract; LPS, lipopolysaccharide; NO, nitric oxide.

Fig. 2 shows the RAW246.7 cell viability after 95ECH extract treatment with or without LPS. The 95ECH extract did not significantly affect macrophages at concentrations of 7.5–120 μg/ml (Fig. 2). Moreover, our results showed that 1 μg/ml of LPS did not change the cell viability of RAW246.7 cells. Up to 240 μg/ml, 95ECH caused a significant decrease in cell viability, and thus subsequent experiments were conducted with 95ECH in the range of 7.5–120 μg/ml.

Neutral red uptake assays were conducted to determine the effects of 95ECH on the phagocytic activity of RAW264.7 macrophages. The phagocytic activity of the LPS-treated group used as a positive control increased to 143.62 compared to the non-treated (control) group (Fig. 2). In contrast, treatment with 15 μg/ml 95ECH significantly inhibited the phagocytosis rate in RAW264.7 cells stimulated with LPS. The 95ECH treatment decreased the phagocytic activity of RAW264.7 macrophages stimulated with LPS in a dose-dependent manner (*p* < 0.05).

The changes in NO production in RAW264.7 macrophages after 95ECH treatment are shown in Fig. 2D. LPS treatment at 1 μg/ml markedly increased nitrite production, whereas 95ECH at 60 μg/ml and 120 μg/ml significantly decreased LPS-stimulated NO production to 24.29 μM and 19.30 μM, respectively.

### Effects of 95ECH from *E. asperula* Leaves on Inflammatory Cytokines and Mediators in LPS-Stimulated RAW264.7 Cells

**Fig. 3:**
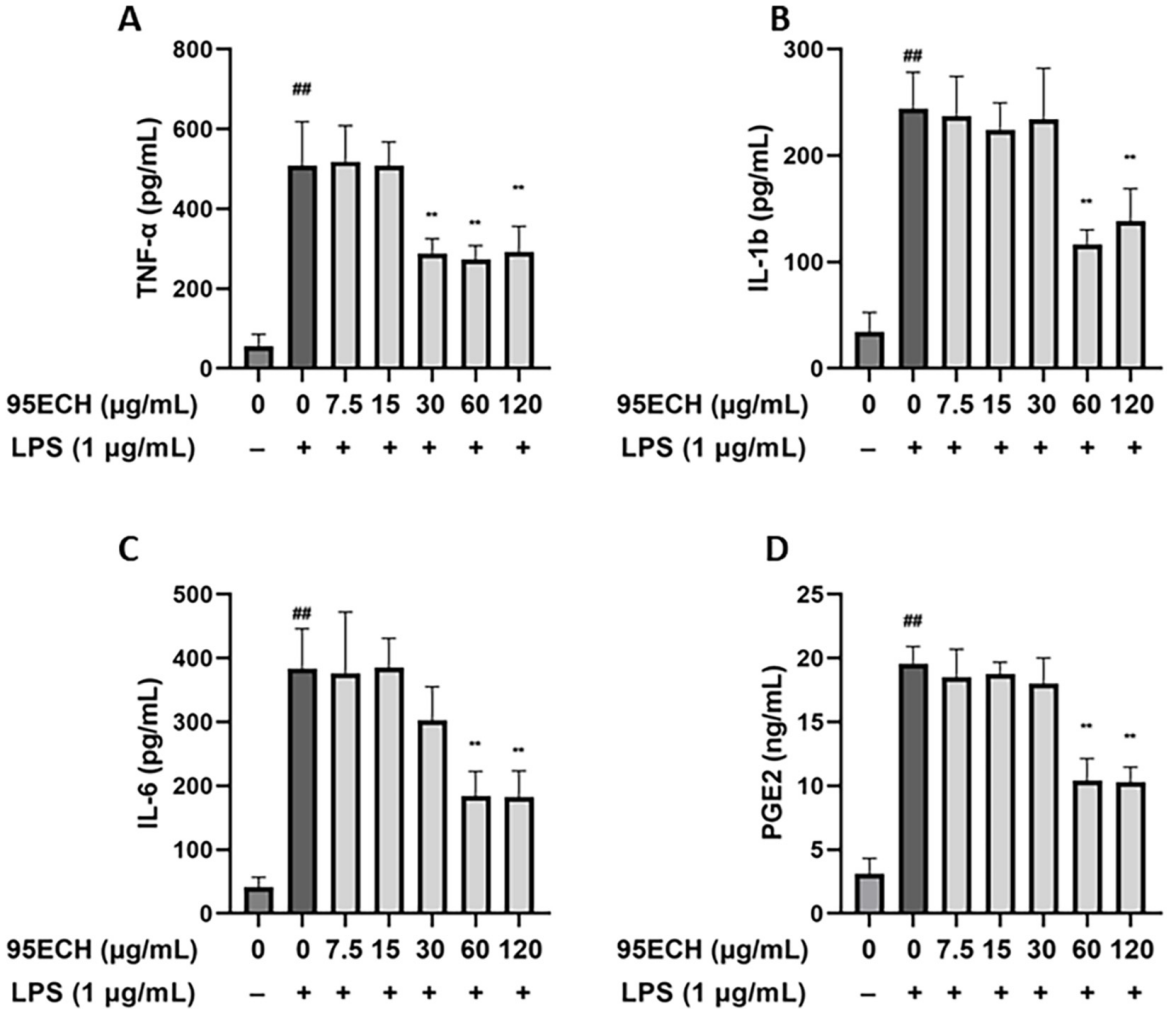
Effects of 95% ethanol extract of *E. asperula* leaves on the secretion of the cytokines. TNF-α (**A**) IL-1β (**B**) IL-6 (**C**) and PGE2 mediator (**D**) on RAW264.7 macrophages. ^##^*p* < 0.01 represents a significant difference compared with the control group; **p < 0.01 represents a significant difference compared with the LPS alone group. 95ECH, 95% ethanol extract; LPS, lipopolysaccharide; NO, nitric oxide; TNF-α, tumor necrosis factor alpha; IL-6, interleukin-6; IL-1β, interleukin-1 beta; PGE2, prostaglandin E2.

In abnormal conditions, activated macrophages secrete diverse inflammation-related cytokines and mediators, such as TNF-α, IL-6, IL-1β, and PGE2 [13]. Therefore, immunoassays were performed to evaluate the effects of 95ECH on the protein production of these compounds. As shown in Fig. 3, LPS promoted the secretion of TNF-α, IL-6, IL-1β, and PGE2, compared with the control group (*p* < 0.01), whereas the secretions were decreased in a dose-dependent manner after 95ECH treatment. The levels of IL-6, IL-1β, and PGE2 were remarkably inhibited at 60 μg/ml 95ECH, whereas TNF-α levels showed a clear reduction at 30 μg/ml 95ECH compared to the control group (*p* < 0.01).

**Fig. 4:**
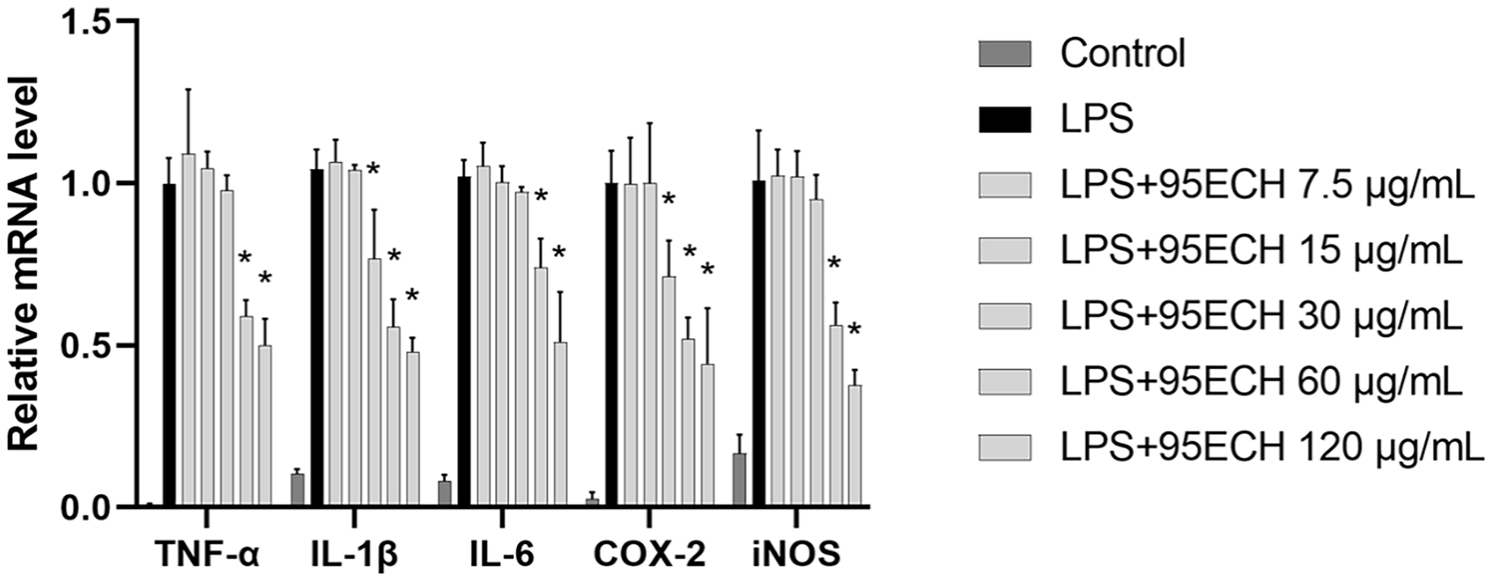
Effects of 95% ethanol extract of *E. asperula* leaves on cytokine gene expression in RAW264.7 macrophages. *p < 0.05 represents a significant difference compared with the LPS alone group. 95ECH, 95% ethanol extract; LPS, lipopolysaccharide; NO, nitric oxide; TNF-α, tumor necrosis factor alpha; IL-6, interleukin-6; IL-1β, interleukin-1 beta; COX-2, cyclooxygenase-2; iNOS, inducible nitric oxide synthase.

Furthermore, the gene expression of these pro-inflammatory cytokines was determined in RAW 264.7 macrophages. As expected, pro-inflammatory cytokines were upregulated in RAW 264.7 macrophages stimulated by LPS (*p* < 0.01) (Fig. 4). LPS increased the expression of cytokines in macrophages, but the expression levels of IF-1b and COX-2 mRNA decreased to 31.23% and 20.77%, respectively, after treatment with 30 μg/mL 95ECH. A similar effect was observed on the mRNA expression of TNF-α, IL-6, and iNOS after treatment with 60 μg/ml 95ECH.

## Discussion

*E. asperula* is an edible plant and rich source of bioactive compounds. Among potential anti-inflammatory compounds, phenols are important plant constituents that inhibit the synthesis of pro-inflammatory mediators and the activity of nitric oxide synthase and cyclooxygenase-2 [14]. In our current work, the total phenolic and flavonoid contents of extracts are consistent with those of previous studies [15]. In addition, ethanol is a classical, non-toxic solvent and Generally Recognized as Safe (GRAS) substance for efficiently extracting phenolic compounds from plants. We found that the yield of *E. asperula* leaf extracts increased with increasing ethanol concentration and reflected the polyphenols extracted, with relation to their solubilization capacity and selectivity. This outcome is consistent with those of other studies suggesting that 95% ethanol greatly promotes the selectivity and efficiency of the extracting process for *Ehretia* plants [16-18].

COX is a type of oxidoreductase enzyme that synthesizes prostaglandins, prostacyclins, and thromboxanes through the conversion of arachidonic acid [19]. Among the three isoforms of COXs, the function of COX-2 is significantly correlated with inflammatory processes, pain, and fever [20]. Therefore, the inhibition of COX-2 is a suitable drug target against inflammatory diseases via reducing prostanoid synthesis. Several flavonoids and flavonoid-rich extracts have been shown to effectively reduce cyclooxygenase-2 activity [21-23]. Samani P., Costa S., and Cai S. [23] showed that blueberry extract was rich in flavonoids that inhibited COX-2 activity with an IC_50_ of 18 μg/mL. In the present study, the different extracts of *E. asperula* leaves exhibited COX-2-inhibiting activity. To the best of our knowledge, it is the first time that such quantitatively characterized, dose-dependent inhibition of COX-2 activity with identified IC_50_ of extracts of *E. asperula* leaves has been described. Our results showed that 95ECH effectively inhibited COX-2 activity at 34.09 μg/ml, suggesting that long-term consumption of *E. asperula* leaves could help improve inflammatory management. We plan to further investigate the use of 95CHE from *E. asperula* in inflammatory cells to explore its anti-inflammatory potential.

Macrophages are important cellular components of the immune response as they possess phagocytic activity and respond to exogenous stimuli. LPS is an inflammatory factor in various cell types and induces an inflammatory reaction via the production of NO and cytokines. First, up to 120 μg/ml 95ECH showed no cytotoxic effects on RAW264.7 macrophages. Importantly, LPS significantly promoted the phagocytic activity of macrophages, whereas 95ECH significantly suppressed this activity (*p*<0.01) in a dose-dependent manner. These results suggest that 95ECH treatment effectively reduced the phagocytosis of LPS-induced RAW264.7 cells, which was an important immunomodulatory effect. Similar to our results, Tian C, Chang Y, Zhang Z, Wang H, Xiao S, Cui C, *et al*. [24] reported that ethanol extracts of *Tribulus terrestris* L. leaves significantly inhibited phagocytosis. Flavonoids are known to have modulating phagocytic activity [25-27].

Another inflammatory evaluation index, NO, is an important molecule produced by activated macrophages and a key chemical indicator of inflammation and inflammatory disease. In inflammatory processes, the production of NO leads to tumor elimination, clearance of invading pathogenic microorganisms, and regulation of the functional activity of immune cell types, but NO excess may adversely affect normal cell function [28]. We observed a reduction of elevated NO content caused by LPS after 95ECH treatment.

Overexpression of cytokine levels in macrophages affects NO production and phagocytic activity. This concept, known as immune-modulating capacity, is used to understand the mode of action of potential therapeutic agents. The production of pro-inflammatory cytokines regulates the proliferation, triggers differentiation, and modifies the function of immune cells [29, 30]. Our results indicated that 95ECH suppressed cytokine production in activated macrophages at the gene level as determined with qRT-PCR, as well as at the protein level as determined with ELISA. We observed a decrease in the gene expression of IL-6, IL-1β, and TNF-α, which play a critical role in the generation and propagation of inflammation. The TNF superfamily is essential in the response to inflammatory signals [31]. TNF-α amplifies inflammation by inducing the secretion of other inflammatory cytokines and activation of T cells. IL-1β is responsible for the induction of fever and stimulates the production of other signaling molecules that link innate and adaptive immune responses such as IL-6 [32]. This is also consistent with the reduction of IL-6 formation, which leads to the induction of acute-phase proteins in the liver and the maturation of B cells in adaptive immunity [33]. In addition, we compared the iNOS mRNA expression in 95ECH-treated groups versus the LPS group. Upon treatment with 95ECH, the iNOS mRNA expression was significantly suppressed. This observation strongly suggests that inhibition of iNOS expression efficiently decreased NO levels. The findings of the present study are also consistent with those of previous reports on medicinal plant extracts [34-36].

PGE2 is a bioactive lipid mediator that plays a critical role in suppressing TNF-α in macrophages [37]. Importantly, its production is managed through transcriptional regulation of the COX-2 gene. For instance, 95ECH strongly suppressed PGE2 via COX-2 inhibition, as evidenced by the reduced gene expression levels of the COX-2 gene. Thus, our study suggests that 95ECH may provide an opportunity to reduce inflammation by suppressing the COX-2/PGE2 and iNOS/NO pathways.

In addition to the few reports on the bioactive effects of *E. asperula* leaf extracts, extracts of some related species of the *Ehretia* genus have been described as having the ability to improve markers of oxidative stress and decrease inflammatory markers, including various cytokines and adhesion molecules. Lim *et al*. [38] showed that the methanol extract of *E. tinifolia* increased antioxidant Nrf2/HO-1 production and inhibited pro-inflammatory NF-κB and MAPKs. Ashagrie *et al*. further examined the effects of *E. tinifolia* extracts on the inflammatory response induced by carrageenan in rats [7]. These observations indicate that bioactive compounds present in *Ehretia* leaves affect plausible pathways for controlling inflammation and therefore require further exploration.

In summary, this study highlights the potential health benefits of *E. asperula* leaves in inflammation, specifically concerning the mechanism of action. Our findings demonstrate the capacity of extracts to inhibit COX-2 activities by chemical composition and in a dose-dependent manner. This study was the first to apply an evidence-based method to verify that 95ECH significantly alleviated inflammation in LPS-activated RAW264.7 cells by inhibiting phagocytosis and NO production. Moreover, the underlying mechanism of 95ECH may involve suppressing the COX-2/PGE2 and iNOS/NO pathways. These data highlighted the importance of *E. asperula* leaves as a novel source of natural compounds with anti-inflammatory activity. However, the main components of 95ECH should be further investigated, and further validation studies in vivo are required.

## Figures and Tables

**Table 1:** Total phenolic and flavonoid contents of *E. asperula* leaf extraction.

Extract	TPC (mg GAE/g extract)	TFC (mg RE/g extract)
n-HCH	111.13 ± 0.08^c^	18.12 ± 0.28^a^
MCH	80.63 ± 2.01^d^	7.85 ± 0.17^d^
95ECH	156.12 ± 0.15^a^	18.33 ± 0.48^a^
80ECH	120.11 ± 0.16^b^	12.45 ± 1.03^b^
50ECH	35.06 ± 1.12^e^	12.06 ± 0.89^c^
WCH	20.56 ± 0.71^f^	8.14 ± 0.34^d^

Values represent the mean of five analytical samples±SD. Different superscript letters (a-e) in the same column represent a statistical difference (*p* < 0.05). n-HCH, hexane extract; MCH, methanol extract; 95ECH, 95% ethanol extract; 80ECH, 80% ethanol extract; 50ECH, 50% ethanol extract; WCH, aqueous extract; COX-2, cyclooxygenase-2; TPC, total phenolics content; TFC, total flavonoid content; GAE, gallic acid equivalents; RE, rutin equivalents.
